# Proenkephalin Levels and Its Determinants in Patients with End-Stage Kidney Disease Treated with Hemodialysis and Peritoneal Dialysis

**DOI:** 10.3390/ijms241915015

**Published:** 2023-10-09

**Authors:** Wiktoria Grycuk, Zuzanna Jakubowska, Jolanta Małyszko

**Affiliations:** Department of Nephrology, Dialysis and Internal Medicine, Medical University of Warsaw, 02-097 Warsaw, Poland; wiktoriagrycuk@gmail.com (W.G.); zuzanna.jakubowska@wum.edu.pl (Z.J.)

**Keywords:** proenkephalin, enkephalins, biomarker, end-stage kidney disease, dialysis, hemodialysis

## Abstract

Recently, proenkephalin A (PENK A) has been shown to reflect glomerular dysfunction and to predict new-onset acute kidney injury and heart failure. While previous studies have investigated PENK A as a biomarker in individuals with preserved renal function, PENK A concentration in patients with end-stage kidney disease (ESKD) was not investigated. Plasma PENK A concentration was assessed in 88 patients with ESKD treated with hemodialysis (HD) or peritoneal dialysis (PD), and its associations with kidney function and heart failure indicators were investigated. In HD patients, the difference in PENK A levels before and after hemodialysis, was measured and further assessed for an association with the type of HD membrane used. PENK A levels did not differ significantly between HD and PD patients. In HD patients, the median PENK A concentration was significantly higher before than after hemodialysis (1.368 vs. 2.061, *p* = 0.003). No correlation was found between PENK A level and urea (*p* = 0.192), eGFR (*p* = 0.922), dialysis vintage (*p* = 0.637), and residual urine output (*p* = 0.784). Heart failure (*p* = 0.961), EF (*p* = 0.361), and NT-proBNP (*p* = 0.949) were not associated with increased PENK A concentration. PENK A does not reflect renal function and cardiac status in patients with ESKD. Further research is required to establish the clinical utility of the new biomarker in patients with impaired kidney function.

## 1. Introduction

In recent years, clinical research on early biomarkers of kidney damage has been intense and proenkephalin A (PENK A) has been identified as a promising filtration biomarker [1]. PENK A is a common precursor of peptides which belongs to the enkephalin family and forms an endogenous opioid system in the central and peripheral nervous system [2]. Enkephalins, including met-enkephalin and leu-enkephalin, act primarily on delta opioid receptors, which exhibit a wide expression in non-neuronal tissues, including the kidney, heart, skeletal muscle, and lungs [2]. In animal studies, delta-opioid receptor activation in the kidney has been shown to increase renal blood flow and diuresis, but its role in regulating renal function in humans has not been well established [3]. The utility of enkephalins in research is limited due to their short half-life and lability in vitro. Unlike enkephalins, PENK A, which reflects the activity of the endogenous opioid system, can be easily measured in plasma and remains stable after collection [4,5].

In several clinical settings, PENK A has been proposed as an accurate marker for the detection of acute kidney injury (AKI) or worsening of renal function (WRF), including chronic kidney disease [6], acute heart failure [7], cardiac surgery [8], and sepsis [9,10,11,12]. In large cohort studies, plasma PENK A concentration has shown a strong correlation with the measured GFR (mGFR) by using iohexol or iothalamate clearance in both a steady renal state and an acute setting [4,9]. In healthy individuals, elevated PENK A concentrations have been shown to be associated with a greater yearly decline of eGFR and to predict the risk of developing chronic kidney disease (CKD) [13,14] during 16.6 years of follow-up. Although these results suggest that PENK A might reflect glomerular dysfunction in individuals with both adequate and reduced filtration rates, and therefore serve as a predictive marker of CKD [4,13], to the best of our knowledge, its concentration in patients undergoing dialysis treatment has not been investigated previously.

More recently, opioid peptides have also emerged as potential modulators of cardiac function [15,16]. Through the activation of G protein-coupled receptors, enkephalins have been shown to inhibit sympathetic stimulation in the autonomic nervous system leading to a decrease in myocardial contractility, heart rate, and blood pressure [15,16]. PENK A levels have been shown to increase in response to cardiac injury both in acute conditions and chronic heart failure (HF) [7,8,17,18]. Elevated levels of PENK A in a large cohort of patients with HF were significantly associated with more advanced stages of the disease and increased mortality [18]. It has been hypothesized that enkephalins might play a compensatory role in HF explained by its cardio-depressive effects, and therefore could be considered to be a disease severity marker [16,18]. However, it remains unclear to what degree proenkephalin concentration can be influenced by confounding factors, including kidney function. As cardiovascular disease is a risk factor for cognitive impairment, a recent study reported associations of PENK A with incident cognitive impairment [19].

While most studies have been focused on investigating PENK A in individuals with AKI or CKD, PENK A levels and its determinants in patients with end-stage kidney disease (ESKD) undergoing dialysis therapy have not yet been established. Since patients with CKD and AKI are susceptible to a rapid decline in kidney function requiring dialysis treatment, further validation of the role of PENK A as an indicator of GFR is required in this setting. Our study aimed to assess the plasma PENK A concentration in patients with ESKD treated with hemodialysis (HD) and peritoneal dialysis (PD) and to investigate its correlation with renal function and heart failure. Additionally, we aimed to determine whether PENK A is removed through hemodialysis membranes.

## 2. Results

### 2.1. Patient Characteristics

The baseline characteristics of the 88 eligible patients are listed in Table 1. The mean age was 60.61 ± 16.43, and 41 (47%) patients enrolled in the study were female. The average eGFR was 5.864 ± 2.374 mL/min/1.73 m^2^ which did not differ between HD and PD patients (5.727 ± 2.012 and 6.273 ± 3.254, *p* = 0.856). In addition, 48 patients (55%) had no or very low (≤500 mL/24 h) residual urine output. Most patients (*n* = 35, 40%) had been receiving dialysis treatment for ≥1 and <5 years, and the remainder of the study population had been treated for <1 year (*n* = 19, 22%), ≥5 and <10 years (*n* = 20, 23%), and ≥10 years (*n* = 14, 16%). The result of the baseline PENK A measurement was available for 86 patients. The median baseline PENK A concentration in the overall population was 1.492 (1.071–4.380) ng/mL. No significant differences were observed between the HD and PD groups (1.368 [1.068–4.030] ng/mL vs. 1.706 [1.211–5.858] ng/mL, *p* = 0.305). In the HD patients, the median PENK A concentration was significantly higher before than after hemodialysis (1.368 [1.068–4.030] ng/mL vs. 2.061 [1.708–3.695] ng/mL, *p* = 0.003).

### 2.2. PENK A and Kidney Function Parameters

**Figure 1:**
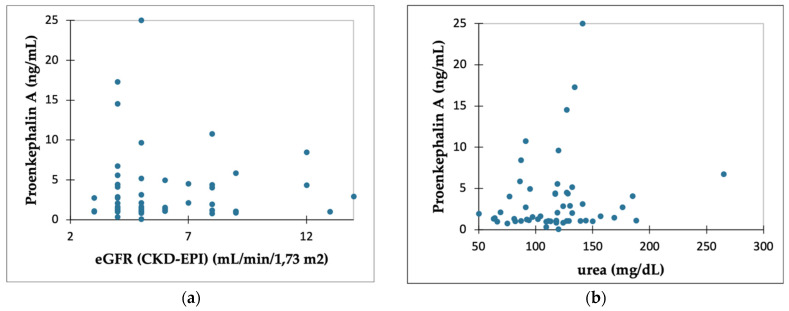

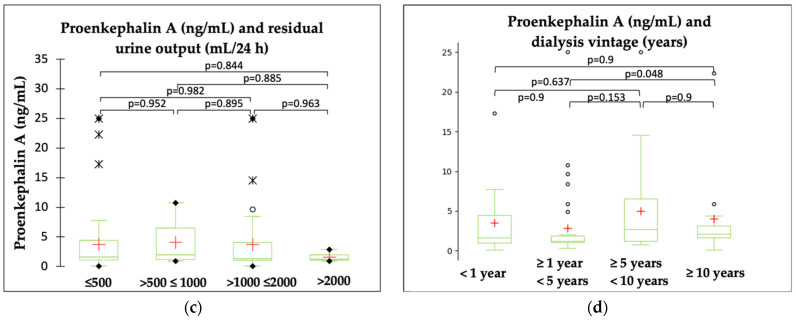
Graphs showing the relationship between PENK A levels and kidney function indicators. No correlation was found between proenkephalin A and (**a**) eGFR (*p* = 0.922) and (**b**) urea (*p* = 0.192); (**c**) there were no significant differences in PENK A levels depending on the residual urine output (*p* = 0.784) and (**d**) dialysis vintage (*p* = 0.067).

Figure 1 shows the results of the correlation analyses which failed to show the link between PENK A levels and eGFR (*p* = 0.922) (a) or urea (*p* = 0.192) (b). PENK A levels did not differ significantly between groups, with varying degrees of residual urine output (*p* = 0.784) (c). Dialysis vintage did not seem to influence PENK A concentrations (*p* = 0.067) (d).

### 2.3. PENK A and HF Indicators

**Figure 2:**
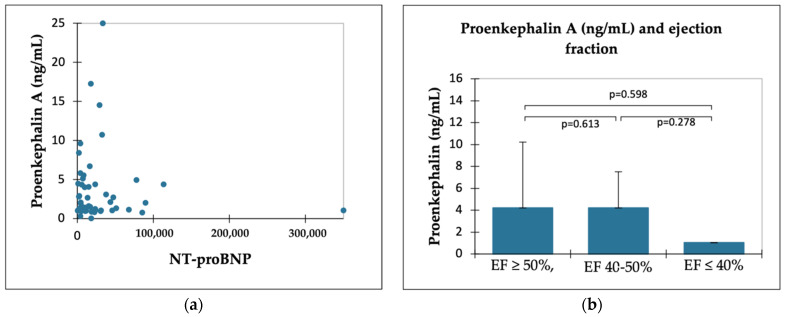

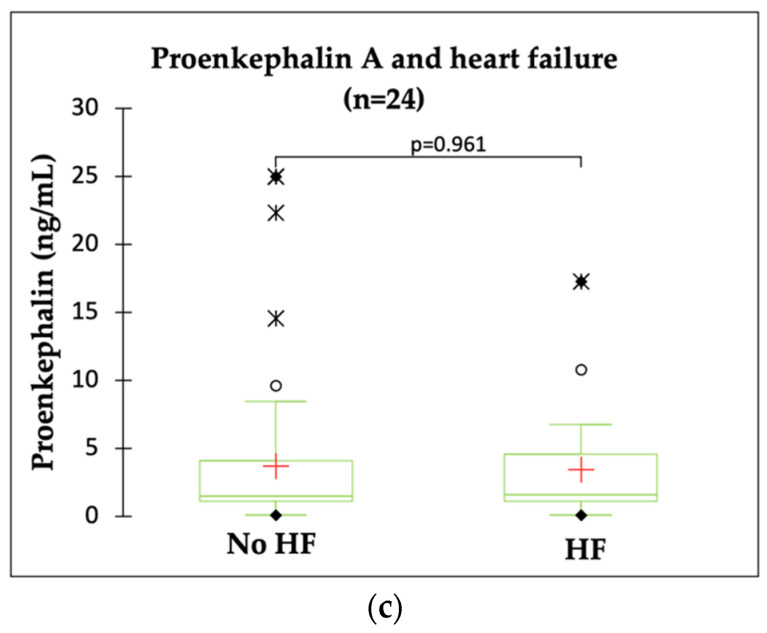
(**a**) No correlation was found between PENK A level and NT–proBNP. The comparison between groups showed no significant differences in PENK A levels depending on (**b**) the range of EF (*p* = 0.361) or (**c**) presence of HF (*p* = 0.961).

The echocardiography report was available in 60 (68%) patients and the NT-proBNP measurement was obtained in 56 (64%) patients. Based on standard criteria (see Section 4.2.) heart failure was diagnosed in 24 (28%) patients. Heart failure with reduced or mildly reduced EF accounted for only eight (33%) cases. The remaining 16 (66%) patients (for whom an echocardiography report was available) were diagnosed with HF with preserved EF. As depicted in Figure 2, Sperman’s rank correlation showed no correlation between PENK A and NT-proBNP (*p* = 0.949) (a). There was no significant difference in PENK A level between patients with varying EF (*p* = 0.361) (b). Similarly, no significant differences in PENK A levels were found between patients with or without HF (*p* = 0.961) (c).

### 2.4. PENK A and Type of Dialysis Membrane

**Figure 3:**
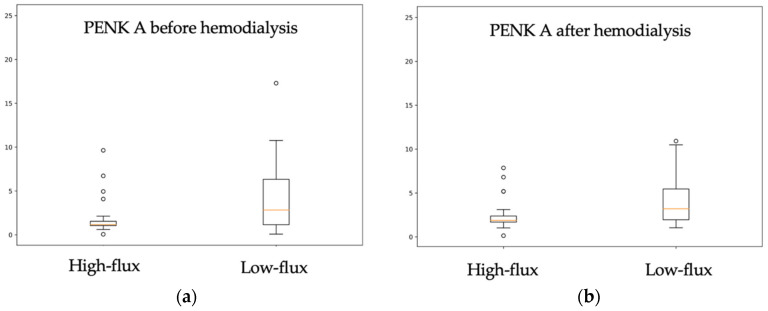
Figure presenting PENK A levels in patients treated with high-flux (boxplot on the left) and low-flux (boxplot on the right) hemodialysis membranes. PENK A concentration was significantly higher in patients using low-flux membrane vs. patients using high-flux membranes, both before hemodialysis (*p* = 0.003) and afterwards (*p* = 0.003): (**a**) PENK A levels in samples drawn directly before hemodialysis; (**b**) PENK A levels after hemodialysis.

**Figure 4:**
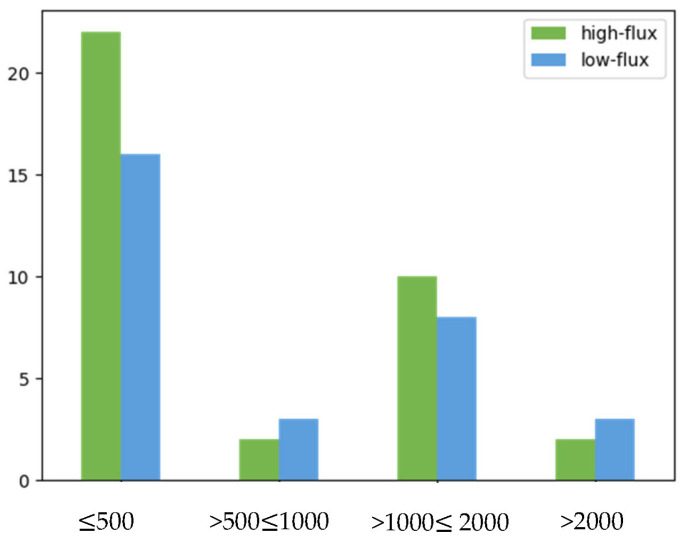
Figure presenting the distribution of residual urine output (mL/24 h) in patients treated with high-flux (green) and low-flux (blue) hemodialysis. No significant differences in residual diuresis were observed between the high-flux and low-flux groups (*p* = 0.793).

In our study, the median PENK A level was significantly lower before hemodialysis than afterwards (1.368 [1.068–4.030] ng/mL vs. 2.061 [1.708–3.695] ng/mL, *p* = 0.003) suggesting that its concentration might depend on fluid removal during the procedure. PENK A concentrations were compared between patients using high-flux (*n* = 36) and low-flux (*n* = 30) membranes. In the high-flux group, the median PENK A levels were 1.174 (IQR 1.061–1.55) ng/mL before hemodialysis and 1.876 (IQR 1.685–2.381) ng/mL after the procedure, and ranged from 2.833 (IQR 1.158–6.333) ng/mL before hemodialysis to 3.205 (IQR 1.954–5.465) ng/mL afterwards in the low-flux group. As shown in Figure 3, PENK A concentrations differed significantly between the two groups; however, higher levels were found in patients treated with low-flux membranes, both before (*p* = 0.003) and after hemodialysis (*p* = 0.003). To determine whether these differences are not influenced by different degrees of residual diuresis, a comparison between high-flux and low-flux groups was performed. As displayed in Figure 4, no difference in residual urine output was found between the high-flux and low-flux groups (*p* = 0.793) (suggesting that residual diuresis does not affect the removal of PENK A in patients undergoing high-flux dialysis).

## 3. Discussion

### 3.1. PENK A as a Biomarker of Kidney Dysfunction

To date, there has been no accurate description of the mechanism by which PENK A levels increase in renal diseases. Glomerular filtration usually allows the passage of small, non-protein-bound molecules into renal tubules; however, there has been no published evidence of PENK A excretion in urine that could validate the data on its renal clearance. The proposed explanation of elevated PENK A levels in renal diseases implicates impaired clearance or upregulation [14,20]. This would imply the accumulation of PENK A in CKD and ESKD, proportionally to the degree of impaired kidney function, whilst our study proved that, in patients with ESKD, PENK A did not reflect kidney function. In this cohort, we did not observe the correlation of PENK A level with creatinine, urea, or residual urine output, which are parameters that represent the reference method to assess the degree of kidney dysfunction.

#### PENK A and Hemodialysis

In our study, PENK A seems to be affected by hemodialysis such that its concentration rises when measured directly after the procedure. Therefore, it might be speculated that PENK A is not removed across hemodialysis membranes and the observed increase in the concentration of PENK A is due to intra-dialytic fluid loss.

Dialysis membranes usually allow the passage of small molecules into dialysate; thus, a post-dialysis decrease in PENK A concentration would be expected. The possible explanation of our finding could be the protein binding of PENK A which stops the molecule’s transfer across hemodialysis membrane. The enkephalins, of which proenkephalin is a precursor, bind to albumin in the blood of mammals, so it can be hypothesized that proenkephalin also interacts with plasma albumin [21]. If our hypothesis is correct, it would also explain why the retention of PENK A is not proportional to the degree of accumulation of low-weigh toxins, including creatinine and urea. Further investigations are, however, required to establish PENK A levels in the dialysate and confirm the albumin-binding.

Previous studies on enkephalins (met-enkephalin and leu-enkephalin) and products of proteolytic cleavage of PENK A, have shown the retention of opioid peptides in ESKD [20,22]. In patients with ESKD receiving hemodialysis treatment, plasma met-enkephalin concentration was four times higher than in healthy subjects and correlated with plasma urea and creatinine [22]. Both standard hemodialysis and hemofiltration caused a slight decrease in met-enkephalin concentration [22]. In contrast, leu-enkephalin concentration was significantly lower in patients with ESKD as compared with healthy control and was not affected by dialysis [22]. Similar results were obtained by Wala-Zielińska et al. [23] who investigated the correlation of CKD-associated pruritus with concentrations of endogenous opioids. In this study, the concentration of met-enkephalin was higher in the group of dialysis patients with pruritus compared to the healthy control group, whereas no significant differences in leu-enkephalin serum levels were found between the study groups.

This is the first study to investigate the precursor molecule, PENK A, in patients with ESKD and to evaluate the impact of hemodialysis on plasma levels of PENK A. The reason for the varying concentrations of opioid peptides in ESKD treated with hemodialysis remains to be elucidated; however, we cannot rule out the possibility that these differences are due to protein binding and the resulting decreased permeability for some molecules.

The fact that PENK A is not removed by hemodialysis calls into question the usefulness of proenkephalin as an everyday use biomarker in AKI. Patients with AKI represent a highly vulnerable group, who are prone to a rapid decline of kidney function, and who might require treatment with hemodialysis. Therefore, a marker that aims to improve the early detection of AKI should also allow monitoring of kidney function during the treatment with hemodialysis.

However, there is no evidence, to date, that PENK A binds to proteins. Given its low molecular weight of 4586 Da, PENK A has been assumed to be freely filtered at the glomerulus [9,13,24]. Thus, we cannot rule out the possibility that PENK A is also removed through the hemodialysis membranes and the concentration rise is due to removal of fluid which exceeds the elimination of the molecules. This could explain why, in our study, we observed higher concentrations in patients dialyzed with low-flux dialyzers, which have smaller pores and lower diffusion potential compared to high-flux membranes. However, as the groups of patients on low-flux and high-flux dialysis were not homogenous, these data should be analyzed with caution.

### 3.2. PENK A in Patients with Heart Failure

The concentration of PENK A has been shown to increase in response to cardiac injury in a variety of disorders, including heart failure [17,18,25]. It has been hypothesized that enkephalins might play a compensatory role in HF explained by its cardio-depressive effects and could, therefore, be considered to be a disease severity marker [16,18]. In a study that evaluated the utility of PENK A in stable ambulatory patients with HF, higher levels of PENK A were associated with lower eGFR and decreased EF [17]. In another cohort of patients with new onset or worsening heart failure, higher PENK A levels were found in patients with more severe HF (reflected by a higher NYHA score and higher natriuretic peptides) and with poorer kidney function (higher creatine and urea levels, lower eGFR) [23]. Importantly, in all of the studies investigating PENK A in patients with HF, PENK A levels were strongly associated with eGFR [17,18,25]. Emmens et al. found that eGFR was the strongest independent predictor of elevated PENK A levels in [18].

Renal dysfunction is a common comorbidity in patients with HF and worsening of the kidney function is associated with increased risk of death and prolonged hospitalization in decompensated HF [26]. Thus, a biomarker which allows monitoring of both kidney and cardiac dysfunction would be desirable. In our study, investigating PENK A in patients with ESKD, no significant differences in PENK A levels were observed between patients with and without HF and with varying degrees of EF. Likewise, no correlation was found between PENK A level and NT-proBNP. As the assessment of the kidney function is desired in all subsets of patients, these results further support our observation that PENK A should not be used as a biomarker in patients with HF and kidney dysfunction, who might potentially require renal replacement therapy.

### 3.3. Limitations of the Study

Patients with end-stage kidney disease represent a highly heterogenous group of patients, making it likely that the results were influenced by a variety of confounding factors. Due to a single-centered design of the study, the results are not necessarily generalizable. Furthermore, the number of patients with diagnosed chronic heart failure was limited in our study and the echocardiographic report was not obtained in all individuals. The gold standard heart failure biomarker, i.e., the NT-proBNP level was not obtained in 32 (36%) patients. Moreover, the measurement of NT-proBNP could likely be influenced by the fluid overload during the inter-hemodialysis period. The available literature suggests that HF coexists with ESKD in approximately 32–44% (PD and HF respectively) [27] of cases which outweighs the percentage of patients with diagnosed HF in our study and may indicate that the number of patients with HF in our cohort was underevaluated.

Despite the fact that anuria is a typical finding in patients with ESKD, it should also be noted that more than half of all patients had no or very low (55%) residual diuresis. Assuming that PENK A is freely filtered at the glomerulus, varying degrees of residual kidney function could have contributed to the retention of PENK A.

In summary, unlike other studies which support the link between PENK A levels with steady-state renal function, we did not find a correlation between PENK and simultaneously drawn creatinine or urea levels and eGFR. Moreover, our study suggests that PENK A is not dialyzable. These findings raise questions about the value of PENK A as a marker of kidney function, particularly when used in a vulnerable subset of patients with AKI or CKD who might require renal replacement therapy.

## 4. Materials and Methods

### 4.1. Patients

We identified 101 patients treated with dialysis in our center between May and June 2022. The inclusion criteria involved: (1) 18 years of age or older, (2) HD or PD treatment for at least 90 days, (3) ability to provide informed consent. A total of 88 patients undergoing HD (*n* = 66) or PD (*n* = 22) who met the inclusion criteria were enrolled in this cross-sectional study. Patients with decompensated heart failure and patients receiving dialysis treatment in the intensive care unit were excluded. All patients received either standard continuous ambulatory and automated PD or HD two (*n* = 9), three (*n* = 55), or four (*n* = 2) times per week using high flux (*n* = 36) and low flux (*n* = 30) membranes.

This study received approval from the Medical University of Warsaw Ethics Committee (no. KB/131/2021, issued on 6 September 2021) and all patients provided their written, informed consent to participate in the study.

### 4.2. Methods

The blood samples for PENK A determination were collected directly before and at the end of a hemodialysis session in HD patients. In PD patients, the samples were drawn at a single time point on regular outpatient visits. The blood samples were collected in tubes containing EDTA-Na and directly centrifuged at 4 °C for 15 min. The obtained plasma was transferred into 1.5 mL microfuge tubes and stored at −80 °C until assay. The samples for determination of serum creatinine, urea, and NT-proBNP levels were drawn at the time of collection of PENK A, and measurements were performed by using standard procedures of the hospital’s central laboratory. Demographic (gender, age) and clinical data (comorbidities, years on dialysis, and residual urine output) were collected from the patients’ histories and recorded in the clinical database. The estimated glomerular filtration rate (eGFR) was calculated by using the creatinine-based 2009 Chronic Kidney Disease Epidemiology Collaboration (CKD-EPI) equation (including serum creatinine, age, gender, and race). The ejection fraction (EF) was estimated by using the standard formula on echocardiography. According to the 2021 Guidelines of European Society of Cardiology, the diagnostic criteria for chronic heart failure involved the presence of clinical signs or symptoms, objective measures of cardiac dysfunction on echocardiography, and elevated plasma concentration of natriuretic peptides. HF was classified based on echocardiographic results into HF with preserved, mildly reduced, or reduced EF if EF was ≥50%, 40–50%, or ≤40%, respectively.

### 4.3. Measurements

The plasma samples were distributed in 96-well plates. The concentration of PENK A was measured by using the Human (PENK A) Elisa Kit (Shanghai SunRedBio, Shanghai, China), a double-antibody sandwich enzyme-linked immunosorbent assay with monoclonal antibodies specific to the PENK A peptide. The standards (PENK A peptide) and samples were incubated in tubes with the detector antibody, and the tubes were washed, and bound chemiluminescence was detected with a luminometer. The lower limit of detection was 0.041 ng/mL, and the measurement range was 0.05–10 ng/mL. According to the manufacturer’s protocol, the samples were run in duplicate. PENK A assay intra- and inter-assay CV were below 9%.

### 4.4. Statistical Analyses

Statistical analyses were performed by using XLSTAT 2022 version 4.1 and STATA software, and *p*-values <0.05 were considered to represent statistical significance. Continuous variables are described as means ± standard deviation (SD) or medians and interquartile range (IQR) unless stated otherwise. Categorical data are described as numbers and percentages. The following continuous variables were categorized based on established cut-off values: residual diuresis (≤500 mL/24 h, >500 and ≤1000 mL/24 h, >1000 and ≤2000 mL/24 h, >2000 mL/24 h), dialysis vintage (<1 year, ≥1 and <5 years, ≥5 and <10 years, ≥10 years), ejection fraction (<40%, 40–50%, >50%). Comparisons between the (two or more) groups were done by using the Kruskal–Wallis test or the Mann–Whitney U test. Spearman rank correlation was used to examine the correlation between two variables.

## 5. Conclusions

Further validation of the role of PENK A as a measure of kidney function is, however, required. Additionally, investigations are required to establish PENK A excretion in urine that would validate the data on its renal clearance and the possibility of using it as a biomarker of kidney injury. Moreover, it needs to be determined whether PENK A is removed through different dialysis membranes and might, therefore, be considered to be uremic toxin in subjects undergoing renal replacement therapy. At present, utility of PENK A as a biomarker of kidney function poses more questions than answers.

## Figures and Tables

**Table 1:** Baseline characteristics of the study group.

	Overall (*n* = 88)	HD (*n* = 66)	PD (*n* = 22)	*p* Value
Age (years)	65 (51–72)			
Female, n (%)	41 (47)	26 (39)	15 (68)	
eGFR (CKD-EPI) (creatinine-based)	5 (4–7)	5 (4–7)	5 (4–8)	0.856
Plasma PENK A (ng/mL)	1.492 (1.071–4.380)	1.368 (1.068–4.030) *n* = 65 *	1.706 (1.211–5.858) *n* = 21 *	0.305
Dialysis vintage, *n* (%)				
<1 year	19 (22)	17 (26)	2 (9)	
≥1 and <5 years	35 (40)	27 (41)	8 (36)	
≥5 and <10 years	20 (23)	13 (20)	7 (32)	
≥10 years	14 (16)	9 (14)	5 (23)	
Residual diuresis (mL/24 h), n (%)				
≤500	49 (56)	38 (58)	11 (50)	
>500 and ≤1000	7 (8)	5 (8)	2 (9)	
>1000 and ≤2000	26 (30)	18 (27)	8 (36)	
>2000	6 (7)	5 (8)	1 (5)	
Heart failure, *n* (%)	24 (27)	23 (35)	1 (5)	
Hypertension, *n* (%)	77 (88)	60 (90)	17 (77)	
Coronary artery disease, *n* (%)	22 (25)	22 (33)	0	
Diabetes, *n* (%)	31 (35)	26 (40)	5 (23)	
NT-proBNP (ng/L)	14,100 (52,000–30,828)	16,164 (7196–32,586) *n* = 40	8270 (3784– 21,231) *n* = 16	0.059

PENK A, proenkephalin A; NT-proBNP, N-terminal pro-B-type natriuretic peptide. Note: Categorical data are presented with percentage (numbers). Continuous variables are presented with interquartile ranges (IQR). * In the remaining two cases, PENK A concentration was not measured due to the sample hemolysis.

## Data Availability

The datasets generated and analyzed in the current study are available from the corresponding author on reasonable request.
